# Two-Year Morbidity–Mortality and Alternatives to Prolonged Breast-Feeding among Children Born to HIV-Infected Mothers in Côte d'Ivoire

**DOI:** 10.1371/journal.pmed.0040017

**Published:** 2007-01-16

**Authors:** Renaud Becquet, Laurence Bequet, Didier K Ekouevi, Ida Viho, Charlotte Sakarovitch, Patricia Fassinou, Gédéon Bedikou, Marguerite Timite-Konan, François Dabis, Valériane Leroy, ANRS 1201/1202 Ditrame Plus Study Group

**Affiliations:** 1 Institut National de la Santé et de la Recherche Médicale Unité 593, Institut de Santé Publique Epidémiologie et Développement, Université Victor Segalen, Bordeaux, France; 2 Projet Agence Nationale de Recherches sur le Sida 1201/1202 Ditrame Plus, Programme PAC-CI, Centre Hospitalier Universitaire de Treichville, Abidjan, Côte d'Ivoire; 3 Service de Pédiatrie, Centre Hospitalier Universitaire de Yopougon, Abidjan, Côte d'Ivoire; National Institute of Child Health and Human Development, United States of America

## Abstract

**Background:**

Little is known about the long-term safety of infant feeding interventions aimed at reducing breast milk HIV transmission in Africa.

**Methods and Findings:**

In 2001–2005, HIV-infected pregnant women having received in Abidjan, Côte d'Ivoire, a peripartum antiretroviral prophylaxis were presented antenatally with infant feeding interventions: either artificial feeding, or exclusive breast-feeding and then early cessation from 4 mo of age. Nutritional counseling and clinical management were provided for 2 y. Breast-milk substitutes were provided for free. The primary outcome was the occurrence of adverse health outcomes in children, defined as validated morbid events (diarrhea, acute respiratory infections, or malnutrition) or severe events (hospitalization or death). Hazards ratios to compare formula-fed versus short-term breast-fed (reference) children were adjusted for confounders (baseline covariates and pediatric HIV status as a time-dependant covariate). The 18-mo mortality rates were also compared to those observed in the Ditrame historical trial, which was conducted at the same sites in 1995–1998, and in which long-term breast-feeding was practiced in the absence of any specific infant feeding intervention. Of the 557 live-born children, 262 (47%) were breast-fed for a median of 4 mo, whereas 295 were formula-fed. Over the 2-y follow-up period, 37% of the formula-fed and 34% of the short-term breast-fed children remained free from any adverse health outcome (adjusted hazard ratio [HR]: 1.10; 95% confidence interval [CI], 0.87–1.38; *p* = 0.43). The 2-y probability of presenting with a severe event was the same among formula-fed (14%) and short-term breast-fed children (15%) (adjusted HR, 1.19; 95% CI, 0.75–1.91; *p* = 0.44). An overall 18-mo probability of survival of 96% was observed among both HIV-uninfected short-term and formula-fed children, which was similar to the 95% probability observed in the long-term breast-fed ones of the Ditrame trial.

**Conclusions:**

The 2-y rates of adverse health outcomes were similar among short-term breast-fed and formula-fed children. Mortality rates did not differ significantly between these two groups and, after adjustment for pediatric HIV status, were similar to those observed among long-term breast-fed children. Given appropriate nutritional counseling and care, access to clean water, and a supply of breast-milk substitutes, these alternatives to prolonged breast-feeding can be safe interventions to prevent mother-to-child transmission of HIV in urban African settings.

## Abstract 2

Given appropriate nutritional counseling and care, access to clean water, and supply of breast milk substitutes, replacing prolonged breast-feeding with formula-feeding appears to be a safe intervention to prevent mother-to-child transmission of HIV in this setting.

## Editors' Summary

**Background.:**

The HIV virus can be transmitted from infected mothers to their babies during pregnancy and birth as well as after birth through breast milk. Mother-to-child transmission in developed countries has been all but eliminated by treatment of mothers with the best available combination of antiretroviral drugs and by asking them to avoid breast-feeding. However, in many developing countries, the best drug treatments are not available to mothers. Moreover, breast-feeding is generally the best nutritional choice for infants, especially in areas where resources such as clean water, formula feed, and provision of healthcare are scarce. And even if formula feed is available, formula-fed babies might be at higher risk of dying from diarrhea and chest infections, which are more common in infants who are not breast-fed. International guidelines say that HIV-positive mothers should avoid all breast-feeding and adopt formula feeding instead if this option is practical and safe for them, which would require that they can afford formula feed and have easy access to clean water. If formula-feeding is not feasible, guidelines recommend that mothers should breast-feed only for the first few months and then stop and switch the baby to solid food. One of these two alternative options should be feasible in most African cities if mothers are given the right support.

**Why Was This Study Done?:**

Several completed and ongoing studies are assessing the relative risks and benefits of the two recommended strategies for different developing country locations, and this is one of them. The study, the “Ditrame Plus” trial by researchers from France and Côte d'Ivoire, was conducted in Abidjan, an urban West African setting. The goal was to compare death rates and rates of certain diseases (such as diarrhea and chest infections) between babies born to HIV-positive mothers that were formula-fed and those that were breast-fed for a short time after birth.

**What Did the Researchers Do and Find?:**

HIV-positive pregnant women were invited to enter the study, and they received short-term drug treatments intended to reduce the risk of HIV transmission to their babies. Women in the trial were then asked to choose one of the two feeding options and offered support and counseling for either one. This support included free formula, transport, and healthcare provision. Babies were followed up to their second birthday, and data were collected on death rates and any serious illnesses. A total of 643 women were enrolled into the study, and safety data were collected for 557 babies, of whom 295 were in the formula group and 262 were in the short-term breast-feeding group. The researchers corrected for HIV infection in the babies and found no evidence that the risk of other negative health outcomes and death rates was any different between the formula-fed babies and short-term breast-fed babies. Looking specifically at individual diseases, the researchers found that the risks for diarrhea and chest infections were slightly higher among formula-fed babies, but this did not translate into a greater risk of death or worse overall health. They also compared the death rates in this study with some historical data from a previous research project done in the same area on children born to HIV-positive mothers who had practiced long-term breast-feeding. The mother-to-child transmission rate of HIV had been much higher in that earlier trial, but looking only at the HIV-negative children, the researchers found no difference in risk for death or serious disease between the formula-fed or short-term breast-fed babies from the Ditrame Plus trial and the long-term breast-fed babies from the earlier trial.

**What Do These Findings Mean?:**

This study shows that if HIV-positive mothers are well supported, either of the two feeding options currently recommended (formula-only feed, or short-term breast-feeding) are likely to be equivalent in terms of the baby's chances for survival and health. However, women in this study were offered a great deal of support and the findings may not necessarily apply to real-life situations in other settings in Africa, or outside the context of a research project. In addition to routine care after birth, access to better drugs to prevent mother-to-child transmission in developing countries remains an important goal.

**Additional Information.:**

Please access these Web sites via the online version of this summary at http://dx.doi.org/doi:10.1371/journal.pmed.0040017.

- Resources from Avert (an AIDS charity) on HIV and infant feeding.
- Information from the US Centers for Disease Control on mother-to-child transmission of HIV
- Guidelines from the World Health Organization on mother-to-child transmission of HIV
- AIDSMap pages on breast-feeding and HIV
- HIV Care and PMTCT in Resource-Limited Setting contains monthly bulletins and a database devoted to HIV/AIDS infections and prevention of the mother-to-child transmission of HIV
- The Ghent group is a network of researchers and policymakers in the area of prevention of mother-to-child transmission of HIV

## Introduction

In high human immunodeficiency virus (HIV) prevalence resource-constrained settings, HIV-infected pregnant women face a dilemma regarding the feeding practices of their forthcoming infant [1]. Indeed, in sub-Saharan Africa, where breast-feeding is widely practiced and usually prolonged at least 1 y after birth, the overall risk of HIV transmission through breast milk was estimated to be 8.9 new cases per 100 child-years of breast-feeding [2], and was thus responsible for 40% of perinatally acquired HIV infections [3]. On the other hand, in the absence of any specific nutritional counseling and adapted clinical management, nonbreast-fed children have a greater risk of dying from infectious diseases, especially early in infancy [4].

Current United Nations recommendations state that “when replacement feeding is acceptable, feasible, affordable, sustainable and safe, avoidance of all breast-feeding by HIV-infected mothers is recommended. Otherwise, exclusive breast-feeding is recommended during the first months of life and should then be discontinued as soon as it is feasible” [5]. Given the necessary support, complete avoidance of breast-feeding or exclusive breast-feeding with early cessation are conceivable nutritional interventions in urban African settings [6]. According to several African studies, the combined promotion of exclusivity of breast-feeding with early cessation could indeed reduce the cumulative risk of postnatal transmission while keeping the benefits of breast-feeding during the first months of life [7–10]. On the other hand, the postnatal risk of HIV transmission no longer exists when breast-feeding exposure is avoided [11].

However, little is known about the safety of these interventions. Compared to unrestricted breast-feeding, complete avoidance of breast-feeding was shown to be safe in an African clinical trial allocating infant feeding practices at random: morbidity and mortality were similar over a span of 2 y in breast-fed and formula-fed children [12]. But so far, child morbidity and mortality have never been studied in real-life situations in which HIV-infected pregnant women are able to choose either to breast-feed for a short period or to formula-feed, while being supported in their choice and counseled accordingly.

The primary objective of this study was to assess the 2-y morbidity and mortality among short-term breast-fed and formula-fed children born to HIV-infected mothers in an urban West African setting with access to clean water. The secondary objective was to assess the 18-mo mortality among children exposed to these alternatives to prolonged breast-feeding and in long-term breast-fed children included in an historical cohort without infant feeding intervention.

## Methods

The ANRS 1202/1202 Ditrame Plus study was an open-labeled cohort, based on patients attending community-run health facilities in Abobo and Yopougon, the two most-densely populated districts of Abidjan, the economic capital of Côte d'Ivoire. In this setting, HIV prevalence was around 11% among pregnant women in 2002 [13], municipal water is of generally good quality [14], and breast-feeding is widely practiced long term [15,16].

The Ditrame Plus study was granted ethical permission in Côte d'Ivoire from the ethical committee of the National AIDS Control Programme, and in France from the institutional review board of the French Agence Nationale de Recherches sur le Sida (ANRS).

### Inclusion Procedures and Research Design

The inclusion procedures and research design undertaken in the Ditrame Plus study were described in detail in previous publications [17,18]. Briefly, from March 2001 to March 2003, any pregnant woman aged 18 y and over, diagnosed as HIV infected within one of the selected community-run health facilities, was proposed for entry into the study.

Women included were systematically presented with both peripartum antiretroviral and postpartum nutritional interventions to prevent mother-to-child transmission of HIV. First, they received a short peripartum drug combination of zidovudine with or without lamivudine and nevirapine single dose [17]. Second, they were systematically and antenatally proposed to practice either complete avoidance of breast-feeding or exclusive breast-feeding with early cessation from the fourth month. Replacement feeding from birth or from breast-feeding cessation until 9 mo of age, as well as the material needed, were provided free of charge. In all cases, the staff supported the choice expressed by the women and counseled them accordingly [18].

### Follow-up Procedures and Data Collection

Two centers were exclusively dedicated to the follow-up of the mother–infant pairs. From birth up to the second birthday, 19 visits were scheduled on study sites for clinical, nutritional, psychosocial, and biological follow-up of both mothers and infants. Mother–infant pairs were seen at birth, 48 h after delivery, weekly until age 6 wk, monthly until age 9 mo, and every 3 mo until the second birthday. At each contact, the medical staff documented clinical events that occurred in children since the last visit. At each scheduled visit, infant feeding practices were recorded via structured questionnaires [19]. Patients who did not keep scheduled appointments were traced and encouraged to return to the study sites.

At each scheduled visit, anthropometric measurements, including height and weight, were taken by trained staff according to standard procedures [20].

Infant feeding counseling was made available at study sites whenever needed [18]. Children requiring intravenous treatment were managed at the day-care hospital units linked to the study sites. For life-threatening diseases or diseases requiring overnight care, children were immediately referred to the pediatric unit of the University Hospital of Yopougon. All transport costs were reimbursed, and all care expenses related to any clinical event were entirely supported by the project.

### Pediatric Diagnosis of HIV Infection

Blood samples were collected at day 2, weeks 4, 6, and 12, and then every 3 mo until 18 mo of age or until 2 mo after complete cessation of breast-feeding if the child was ever breast-fed. A serology examination was systematically performed at age 18 mo in all children. Pediatric HIV infection was defined as a positive RNA PCR at any age or positive HIV serology if aged 18 mo or more [21]. HIV-infected children received cotrimoxazole chemoprophylaxis from the time of their HIV diagnosis.

### Clinical Definitions

Special attention was given to the collection of data on child morbidity potentially linked to inadequate infant feeding practices: diarrhea, acute respiratory infections, or malnutrition. During the study, all reports of potential outcomes were referred for independent review and classification by an event documentation committee unaware of the child's feeding practices. This committee used all clinical information available, including hospital records if the child had been hospitalized. The following definitions were used to validate morbidity. Diarrhea was defined as the passage of three or more loose or watery stools during a 24-h period for at least 2 d, or any reported diarrhea associated with at least one clinical sign of dehydration, or any reported diarrhea requiring care and followed by at least a second consultation for the same reason during a 72-h period. A diagnosis of acute respiratory infection was made if the child presented a cough, fever (axillary temperature greater than 37.5 °C), and focal pulmonary findings on physical examination. A diagnosis of malnutrition was considered when the child presented with growth faltering (no change or a decrease in measurements on growth charts from one visit to another) and was referred to the nutritionists to receive appropriate nutritional care, including provision of protein-enriched food.

In case of child death, verbal autopsies were systematically conducted by trained psychosociologists to assign a possible cause of death [22]. The potential contributing causes of death were independently assessed by two pediatricians, unaware of the child's feeding practices, on the basis of all the clinical information collected (including hospital records) and the verbal autopsy. In case of conflicting diagnosis between the two pediatricians, the opinion of a third one was sought. Causes of death were codified using the 10th revision of the International Statistical Classification of Diseases and Related Health Problems [23].

### Study Outcomes

The primary outcome of the study was the 2-y occurrence in children according to infant feeding practices and HIV status of adverse health outcomes: any severe event (death, or hospital admission related to any cause or in any location) or validated morbid event as defined above. The two components of this definition were also investigated separately as secondary outcomes (severe events and validated morbidity).

A secondary analysis was performed to compare the 18-mo mortality among children exposed to alternatives to prolonged breast-feeding with the mortality of long-term breast-fed children using the ANRS 049 Ditrame trial. This historical trial was conducted in Abidjan, Côte d'Ivoire, and Bobo-Dioulasso, Burkina Faso, in 1995–1998, and it evaluated the efficacy of zidovudine to reduce mother-to-child transmission of HIV [24,25]. For the present analysis, we have included women from the Ditrame trial recruited in Côte d'Ivoire only. These women were recruited at the same sites and in the same population as the Ditrame Plus study. No strategy was proposed at that time to prevent the postnatal transmission of HIV: infants were long-term breast-fed as they usually are in Abidjan [9].

### Statistical Analysis

All live-born infants were available for analysis. In the case of multiple births, only the first born was included. Live-born infants fed at least once were classified in either the breast-fed or formula-fed group on the basis of the infant feeding practices recorded at the visit 2 d after birth, i.e., according to the feeding practice that had been actually initiated. Infants who died or were lost to follow-up before having been fed at least once were unclassified.

Baseline characteristics were compared between these two groups using the Pearson χ^2^ test or the Fisher exact test to compare categorical variables, and the Mann-Whitney *U* test to compare continuous variables.

Compliance with the infant feeding choice was assessed and defined as follows: breast-feeding mothers were considered noncompliant if they had ceased breast-feeding before the third month, and nonbreast-feeding mothers were considered noncompliant if they had breast-fed at least once over the study period. Total effective follow-up time expressed in person-years was compared to total expected follow-up time in both groups. The causes reported for stopping follow-up before the expected 24 mo were described.

The cumulative probabilities of remaining free from an adverse health outcome, a severe event or a validated morbid event, were compared between short-term breast-fed and formula-fed infants using time-to-failure methods, including the Kaplan-Meier estimation and log-rank testing. Multivariate analysis used Cox proportional-hazard models. This approach allowed for the estimation of HRs for mortality and morbidity between the two groups, with adjustment for pediatric HIV status (time-dependent variable) and other covariates at baseline (maternal education, type of housing, type of water supply, baseline maternal CD4 count, living or not with one's partner, study site, and low birth weight). Incidence rates of diarrhea, acute respiratory infection, and malnutrition were expressed per 100 person-year at risk, according to infant feeding practices and HIV status. Estimates were reported with their 95% confidence intervals (CIs).

All statistical analyses were carried out with the use of SAS software (version 8.2; SAS Institute, http://www.sas.com).

## Results

The cohort profile from acceptance of HIV testing to enrollment in the Ditrame Plus study is described elsewhere [17,18]. Among the 643 HIV-infected pregnant women consecutively enrolled between March 2001 and March 2003, 19 with a nonconfirmed HIV-1 status, or infected with HIV-2 only, were excluded, 44 were lost to follow-up before delivery, and 580 gave birth to 612 children [18]. After exclusion of second- and third-born babies of multiple births, 580 mother–infant pairs were included in the present analysis. Of these, 11 (1.9%) were stillbirths, 11 (1.9%) died within the first 72 h of life without having received any food, and one (0.2%) was lost to follow-up before recording information on first feed. Among the 557 live-born children fed at least once, 295 (53%) constituted the formula-fed group and 262 (47%) the short-term breast-fed group.

### Baseline Study Population Characteristics

Baseline characteristics of mother–infant pairs in the Ditrame Plus short-term breast-fed and formula-fed groups are summarized in Table 1. Compared to breast-feeding mothers, formula-feeding mothers had a significantly higher level of education, were less likely to have cospouses or live-in typical shared housing, and were more likely to have tap water access at home. The other sociodemographic, clinical, and biological characteristics were comparable between the two groups.

**Table 1:**
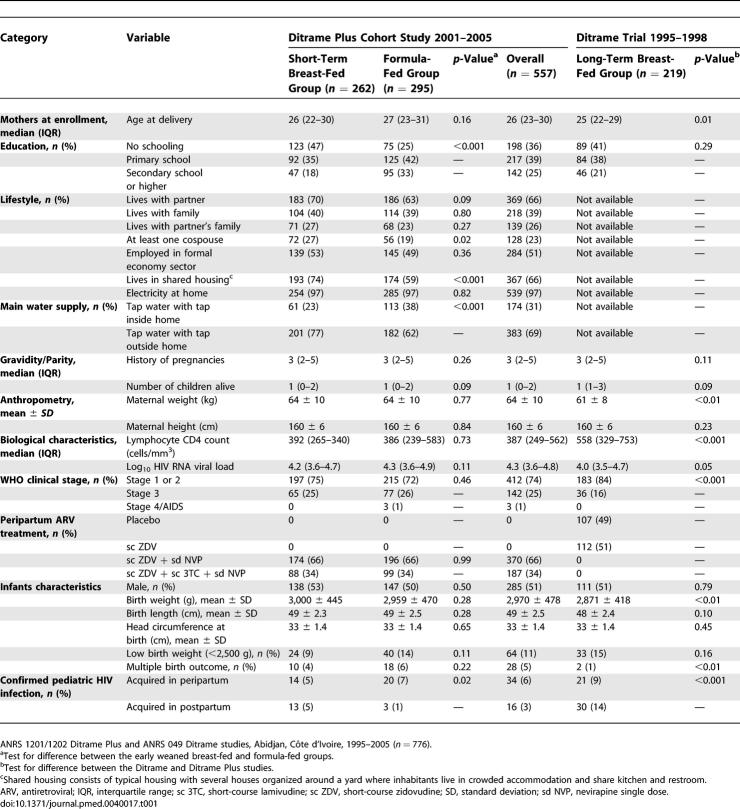
Baseline Characteristics of Mother–Infant Pairs, According to the Feeding Group

### Compliance with the Initial Infant Feeding Maternal Choice

In the breast-feeding group, 24 women (9%) were not compliant with the nutritional intervention agreed upon with the study team because they ceased breast-feeding before age 3 mo and switched to feeding their infants with artificial foods. Among the 262 breast-feeding mothers, complete cessation of breast-feeding occurred a median of 4 mo after delivery (interquartile range [IQR], 3–5 mo). In this group, 91% of the children were breast-fed for at least 3 mo, and 47% were still being breast-fed at age 6 mo. Women were encouraged to practice exclusive breast-feeding, but they failed; instead, most of the infants were predominantly breast-fed during the first 3 mo of life (i.e., children were given small amounts of water or water-based drinks in addition to breast milk) [18].

In the formula-feeding group, 44 women (15%) were noncompliant because they were found to have practiced breast-feeding at least once, 83% of them before their child was 1 mo of age. They thereafter switched to predominant breast-feeding.

### Mother–Infant Pair Follow-up

Total follow-up time was 421 person-years among short-term breast-fed children and 517 person-years among formula-fed children, yielding, respectively, 85% and 92% of expected follow-up times (Table 2). Follow-up was stopped before age 24 mo for 107 children, accounting for 22% of the breast-fed children and 16% of the formula-fed children (*p* = 0.06). For these 107 lost-to-follow-up children, the median age at the end of their observation time was 364 d (IQR, 92–508 d), and was significantly higher in formula-fed (415 d in median, IQR, 260–547 d) than breast-fed children (245 d, IQR, 42–456 d).

**Table 2:**
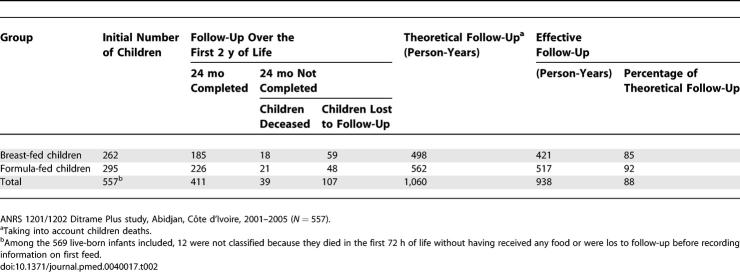
Follow-Up Characteristics among Breast-Fed and Formula-Fed Children

The reported causes for stopping follow-up before age 24 mo did not differ between the two groups and were as follows: relocation outside the Abidjan limits (40%), refusal linked to the study protocol (too many scheduled visits or blood samples collected, 15%), family problems (mother ill or deceased, child with the father outside Abidjan, or widowhood, 9%), fear of stigmatization linked to the study participation (7%), and unspecified for the remainder (29%).

### Occurrence of Adverse Health Outcomes

**Figure 1:**
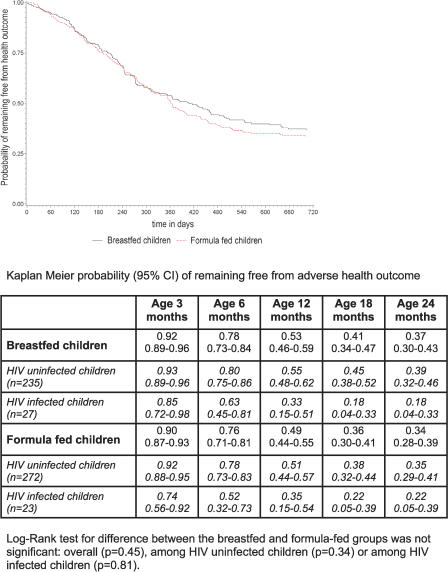
Two-Year Probability of Remaining Free from Adverse Health Outcome (Hospitalization or Death or Validated Morbidity) According to the Feeding Group and Pediatric HIV Status ANRS 1201/1202 Ditrame Plus study, Abidjan, Côte d'Ivoire, 2001–2005 (*n* = 557).

As shown in Figure 1, 37% of the formula-fed and 34% of the short-term breast-fed children remained free from an adverse health outcome, yielding an unadjusted HR among formula-fed children compared to breast-fed children of 1.09 (95% CI, 0.87–1.35; *p* = 0.43). After adjustment for pediatric HIV status and baseline covariates described in Methods, this HR was 1.10 (95% CI, 0.87–1.38, *p* = 0.43).

In a multivariate analysis, the occurrence of an adverse health outcome was only significantly associated with the diagnosis of pediatric HIV infection (HR, 3.1; 95% CI, 2.3–4.1).

### Occurrence of Severe Events

The overall 2-y probability of survival in the Ditrame Plus study was 90%. Mortality rates did not differ significantly between short-term breast-fed and formula-fed children. Over the 2-y period, short-term breast-fed and formula-fed children were comparable for cause of death; however, the frequency of diarrhea tended to be higher in formula-fed children (*p* = 0.10) (Table 3).

**Table 3:**
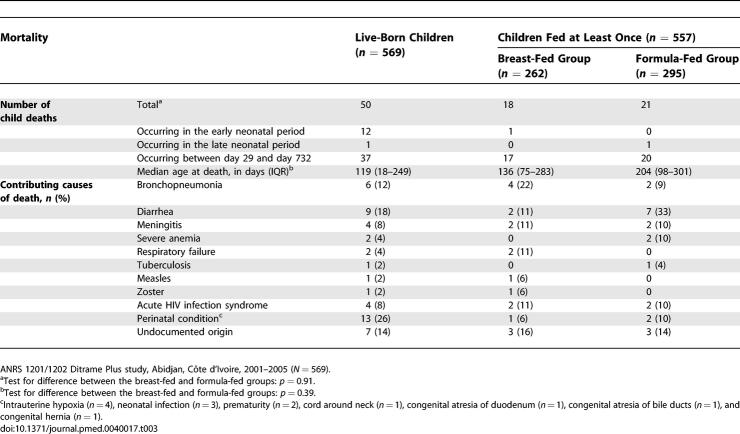
Potential Contributing Causes of Death among Children According to the Feeding Group

**Figure 2:**
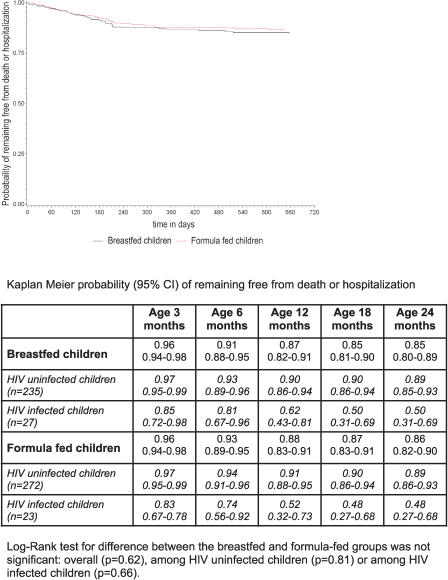
Two-Year Probability of Remaining Free from Severe Events (Hospitalization or Death) According to the Feeding Group and Pediatric HIV Status ANRS 1201/1202 Ditrame Plus study, Abidjan, Côte d'Ivoire, 2001–2005 (*n* = 557).

Among the 557 children, 75 died or were hospitalized: 39 were in the formula-fed group and 36 in the breast-fed group. As detailed in Figure 2, the probability of remaining free from hospitalization or death over the first 2-y of life was the same in the two groups, even after adjustment for potential confounders. The unadjusted and adjusted HRs among formula-fed children compared to short-term breast-fed children were 0.89 (95% CI, 0.57–1.40; *p* = 0.62) and 1.19 (95% CI, 0.75–1.91; *p* = 0.44), respectively.

In a multivariate analysis, the occurrence of death or hospitalization was significantly associated with the diagnosis of pediatric HIV infection (HR, 15.2; 95% CI, 9.4–24.6), low birth weight (HR, 1.8; 95% CI, 1.1–3.3) and mother's illiteracy (HR, 2.0; 95% CI, 1.2–3.2), but not with infant's mode of feeding (HR, 1.2; 95% CI, 0.7–1.9). Very similar results were obtained when performing this analysis among HIV-uninfected children alone.

### Occurrence of Validated Morbidity

**Figure 3:**
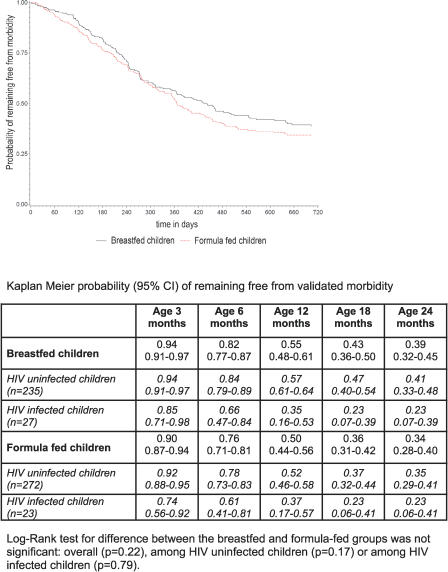
Two-Year Probability of Remaining Free from Validated Morbidity (Diarrhea, Acute Respiratory Infection, or Malnutrition) According to the Feeding Group and Pediatric HIV Status ANRS 1201/1202 Ditrame Plus Study, Abidjan, Côte d'Ivoire, 2001–2005 (*n* = 557).

Over the 2-y period, 36% of the children remained free from diarrhea, acute respiratory infection, or malnutrition (Figure 3). This percentage was slightly higher in formula-fed compared to short-term breast-fed children, but the difference never reached statistical significance, even after adjustment for potential confounders. The unadjusted and adjusted HRs among formula-fed children compared to breast-fed children were 1.15 (95% CI, 0.92–1.43; *p* = 0.22) and 1.16 (95% CI, 0.92–1.46; *p* = 0.21), respectively.

The incidence rates of diarrhea, acute respiratory infection, and malnutrition are reported in Table 4. Compared to short-term breast-fed children, the incidences of diarrhea and acute respiratory infection were higher among formula-fed children (27 versus 22 cases and nine versus six cases per 100 person-years, respectively), yielding adjusted HRs of 1.4 and 1.7 (*p* = 0.03 and *p* = 0.04, respectively). The incidence of malnutrition tended to be higher in breast-fed than formula-fed children (14 versus 11 cases per 100 person-years), but this difference was not statistically significant, even after adjustment.

**Table 4:**
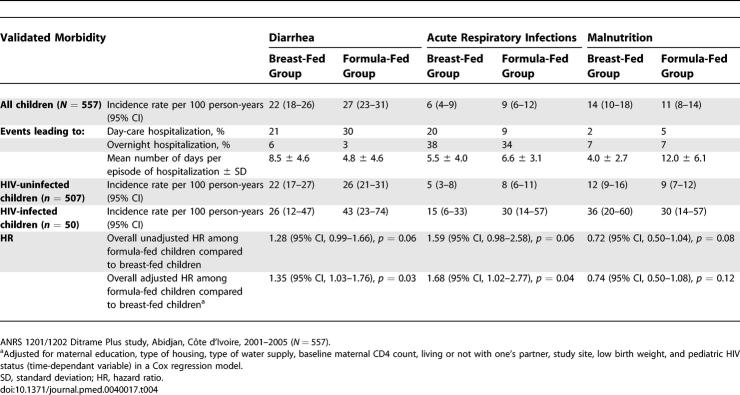
Incidence Rates of Validated Morbidity According to the Feeding Group and Pediatric HIV Status

### Comparison of Mortality Rates with Long-Term Breast-Fed Infants

In 1995–1998, 240 women delivered 243 children in the Abidjan site of the Ditrame trial. After exclusion of three second-born babies of multiple births, four stillbirths, four children who died within the first 72 h of life without having received any food, three lost to follow-up before recording information on first feed, and ten nonbreast-fed children, 219 long-term breast-fed children were included in the present analysis.

Baseline characteristics of these mother–infant pairs are presented in Table 1 and compared with the patients of the Ditrame Plus study. Women used for historical comparison were significantly younger, had higher CD4 counts, and were more likely to be at World Health Organization (WHO) clinical stage 1–2, whereas their children had lower birth weight than in the Ditrame Plus study.

The median duration of breast-feeding in the Ditrame trial was 8 mo (IQR, 6–10 mo), and 80% of the children were still being breast-fed at age 6 mo.

As detailed in Table 5, the overall 18-mo probability of survival was significantly higher in the Ditrame Plus study than in the Ditrame trial: unadjusted HR of 2.24 (95% CI, 1.41–3.56; *p* < 0.001). But the 18-mo probability of survival was similar among formula-fed and short-term and long-term breast-fed HIV-uninfected children.

**Table 5:**
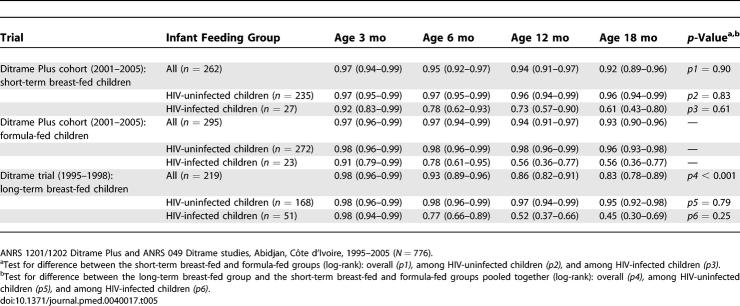
The 18-Mo Probability of Survival (95% CI) According to the Feeding Group and Pediatric HIV Status

In a multivariate analysis, the occurrence of death was significantly associated with the diagnosis of pediatric HIV infection (HR, 14.4; 95% CI, 8.5–25.5), low birth weight (HR, 1.9; 95% CI, 1.1–3.3), mother's WHO clinical stage 3 (HR, 1.7; 95% CI, 1.1–2.9), mother's illiteracy (HR, 1.7; 95% CI, 1.1–2.8), but not with infant's mode of feeding (long-term breast-fed versus short-term breast-fed and formula-fed: HR, 1.3; 95% CI, 0.8–2.1).

## Discussion

In this large prospective cohort study, we found no difference in 2-y rates of adverse health outcomes between early weaned breast-fed and formula-fed children born to HIV-infected mothers. Moreover, the 2-y probabilities of remaining free from severe events (hospitalization or death) and from morbidity validated by an independent committee were comparable in these two groups. However, compared to short-term breast-fed children and after adjustment for potential confounders, the formula-fed ones had a slightly increased risk of diarrhea or acute respiratory infections, but this difference materialized into neither differences in malnutrition rates nor hospitalization or death rates.

Another important issue was whether these two modified infant feeding practices were safe as opposed to the standard, more prolonged breast-feeding. For that purpose, we compared 18-mo mortality among these short-term breast-fed and formula-fed children with the mortality observed in long-term breast-fed children within a historical trial conducted in the same population. No excess in mortality was observed in children exposed to alternatives to prolonged breast-feeding when taking into account HIV status: an overall 18-mo probability of survival of 96% was observed among HIV-uninfected short-term and formula-fed children, which was similar to the 95% probability observed in the long-term breast-fed ones.

At first glance, a randomized clinical trial allocating infant feeding modalities at random would have been the ideal design for investigating adverse health outcomes. We, however, believe the way of feeding one's forthcoming child has to follow an informed and reasoned choice, and thus depends on individual situations. The choice of the infant feeding modality was thus left to the mother in a real-life situation in which alternatives to prolonged breast-feeding were available. The corollary of this nonrandomized design was that breast-feeding women of the Ditrame Plus study were different from those who did not breast-feed. The comparison between the feeding groups could have thus been biased by these differences. The breast-feeding group indeed had lower maternal education, lived in more crowded housing, and was less likely to have in-house access to tap water. All of these factors could be expected to be associated with greater morbidity and mortality in the breast-fed group, but all our analyses adjusted for these potential confounders to minimize this bias.

The Ditrame trial was used as a group of comparison because no alternatives to prolonged breast-feeding had been proposed within that study. The strength of this strategy was that the women had been recruited at the same sites, with the same criteria, and in the same population as the Ditrame Plus study, and both of these studies were coordinated by the same study group. Morbidity data had not been collected and validated as in the Ditrame Plus study, however, limiting our present analysis to mortality. Because the Ditrame trial and the Ditrame Plus study were performed in the same setting by the same team, we cannot exclude the possibility that the medical team was less knowledgeable about managing infants born to HIV-infected women in the earlier period of the research program. We also acknowledge that there may have been confounding factors that had not been taken into account by our design, but we consider that the most important ones were controlled for.

We had previously reported that the women included in the Ditrame Plus cohort were representative of the general population of pregnant women in Abidjan because they had been prenatally recruited among all attendees of community-run health facilities located in poor areas, with no other selection criteria than being HIV infected and at least 18 y old, and having accepted the study protocol [18]. All the women included in the study had access to tap water. But because two-thirds of them lived in typical shared housing, the tap was mainly outside the home. It had been previously reported that the quality of municipal water in Abidjan was good, but that household water storage was a common practice that contributed to contaminated drinking water [14]. Within our study, women were encouraged to avoid water storage, but one-third of them reported having given stored water to their child [26]. Such a practice might have had adverse consequences on infant health.

Emphasis was made in the study protocol on the quality of follow-up of mother–infant pairs. Overall, 88% of the expected follow-up had been completed, and lost-to-follow-up children had participated in the study for a median of 1 y. The reasons for stopping follow-up earlier than expected was recorded for two-thirds of these women, which means that they had come to study sites to explain their intent or that they had been traced at home. In all cases, the vital status of the child was recorded at this last contact. Most of these women were unable to continue their study participation because they moved outside Abidjan to return to the north part of Côte d'Ivoire or to their birth country (mainly Mali or Burkina Faso), which might have been linked to the current political crisis in Côte d'Ivoire. As reported within another study, the follow-up was better among nonbreast-feeding mothers [12]. This could be explained by the mother's sociodemographic characteristics or the health care workers' attitude toward this latter group, and could be a possible source of bias [27].

Given the relatively high standard of care proposed within our study (closed clinical follow-up adapted to the child's age with free provision of care), our primary outcome was the occurrence of adverse health outcomes, defined as morbidity, hospitalization, or death. Emphasis was thus made on the collection and validation of morbidity. The same criteria were used for breast-fed and formula-fed children because the event validation committee was blinded to the exact child feeding practices. Because most of the women in our study were illiterate, we have extended the WHO definition of diarrhea to any diarrhea associated with dehydration or requiring at least a second consultation for the same reason. This may have contributed to a slightly overestimated incidence of diarrhea.

Overall, we believe follow-up quality was high enough and sufficiently unbiased so that estimates of the incidence of adverse health outcomes are adequately made with the same definitions in both groups.

When compared with the Ditrame trial in which no specific infant feeding counseling was made available, the provision of peripartum antiretroviral prophylaxis combined with the promotion of two alternatives to prolonged breast-feeding within Ditrame Plus considerably reduced the number of HIV-infected children: mother-to-child transmission of HIV was significantly reduced with a long-term benefit sustained until age 18-mo. For instance, 18-mo HIV transmission rates as low as 7% (95% CI, 4%–11%) and 6% (95% CI, 2%–10%) were obtained in short-term breast-fed and formula-fed children, respectively, whose mothers had received a peripartum short-course combination of zidovudine and 3TC in addition to single-dose nevirapine [28]. In comparison, the HIV transmission rate was 22% (95% CI, 16%–30%) at age 18 mo among the long-term breast-fed children of the Ditrame trial. Moreover, the mortality among HIV-uninfected children exposed to short-term breast-feeding or formula feeding was similar to the mortality observed among long-term breast-fed uninfected children. Hence, the overall probability of survival was improved among early weaned and formula-fed children in comparison to the long-term breast-fed ones.

Given appropriate nutritional counseling and care, access to clean water, and an adequate supply of breast-milk substitutes, early weaning and formula feeding were not harmful for infant health among HIV-exposed children. Given these constraints, these alternatives to prolonged breast-feeding were not only safe, but socially acceptable and feasible within our population-based study. These results need to be balanced with the evaluation of other outcomes such as the assessment of child growth according to infant feeding practices and maternal perceptions of stigma given different infant feeding strategies.

These findings of the Ditrame Plus study are consistent with a previous clinical trial of randomly allocated infant feeding practices in Kenya, with the preliminary results of a cohort study in Uganda and with a large African pooled analysis [12,29,30]. However, these findings differ from operational research suggesting that formula feeding was associated with higher mortality, morbidity, and stigma in field settings [31,32]. More recently, a clinical trial conducted in Botswana allocated at random 6 mo of breast-feeding plus prophylactic infant zidovudine, or formula feeding plus 1 mo of zidovudine [33]. In that trial, the probability of infant death by month 7 was significantly higher in the formula-fed group than in the breast-fed group (9.3% versus 4.9%; *p* = 0.003), but this difference diminished beyond month 7, such that the time-to-mortality distributions through 18 mo of age were not significantly different (10.7% versus 8.5%; *p* = 0.21).

The access to care, support, and counseling, the provision of the breast-milk substitutes, and the clean water availability, as well as the good follow-up observed within the Ditrame Plus study, would all be expected to lead to more optimal outcomes for alternatives to prolonged breast-feeding in contrast to what was observed in less well-structured or well-supported programs. However, the clean water access, the education level of the mothers, and the economic status of the families included in our cohort appear representative of many urban settings in Africa and lead to a cautious but possible generalization of our results to contexts with appropriate political and structural supports.

In conclusion, we urge the operational implementation in urban African settings of programs aimed at the overall reduction of mother-to-child transmission of HIV. HIV-infected pregnant women could be offered several alternatives to prolonged and predominant breast-feeding so that they could find the one adapted to their individual situation: either complete avoidance of breast-feeding or exclusive breast-feeding with early cessation. The recent rollout in Africa of programs of access to care for people living with HIV could provide a unique opportunity to routinely implement these infant feeding strategies. Both women and children could be given appropriate nutritional counseling and care within these initiatives. Moreover, the clinical support available in such infrastructures could contribute to minimizing infant mortality. The place of heavily subsidized breast-milk substitutes within these programs should, however, be politically discussed. At the same time, more research is needed to improve on safe infant feeding options for resource-constrained settings.

## Supporting Information

Alternative Language Abstract S1 Translation of the Abstract into French by Renaud Becquet (22 KB DOC)

## Abbreviations

ANRS: Agence Nationale de Recherches sur le Sida
CI: confidence interval
HR: hazard ratio
IQR: interquartile range
WHO: World Health Organization
