# Supplementary material for: Two-Year Morbidity–Mortality and Alternatives to Prolonged Breast-Feeding among Children Born to HIV-Infected Mothers in Côte d'Ivoire
Source: PLoS Med. 2007 Jan 16;4(1):e17. doi: 10.1371/journal.pmed.0040017 (PMC1769413; doi:10.1371/journal.pmed.0040017)
Supplement: Supplementary file 1 [file pmed.0040017.sd001.doc]

**Two-year morbidity and mortality**

**and alternatives to prolonged breastfeeding among children born to**

**HIV-infected mothers: ANRS 1201/1202 Ditrame Plus, Abidjan, Côte d’Ivoire**

Renaud BECQUET et al.

French abstract

**Mortalité et morbidité à deux ans chez des enfants nés de mères infectées par le VIH en fonction des pratiques d'alimentation infantile destinées à réduire la transmission postnatale du VIH : le projet ANRS 1201/1202 Ditrame Plus, Abidjan, Côte d'Ivoire**

**Contexte.** Les répercussions à long terme sur la santé infantile des alternatives à l’allaitement maternel prolongé destinées à prévenir la transmission mère-enfant du VIH par l’allaitement maternel sont peu connues à l’heure actuelle.

**Méthodes et résultats.** En 2001-2005, il a été proposé à des femmes enceintes infectées par le VIH de pratiquer une alimentation artificielle ou un allaitement maternel exclusif avec sevrage précoce dès le quatrième mois pour réduire la transmission postnatale du VIH. Les couples mère-enfant ont eu pendant deux ans un suivi nutritionnel et clinique rapproché avec fourniture gratuite du lait maternisé. Le critère de jugement était la survenue de problèmes de santé chez l'enfant : événements morbides (diarrhée, infection respiratoire, malnutrition) ou sévères (hospitalisation ou décès). Les résultats étaient ajustés sur le statut VIH de l'enfant et sur les facteurs de confusion potentiels. La mortalité à 18 mois a été comparée à celle observée historiquement dans l'essai Ditrame conduit sur les mêmes sites en 1995-1998 et où les femmes pratiquaient un allaitement maternel prolongé.

Parmi les 557 enfants inclus, 262 furent allaités pendant quatre mois en médiane et 295 furent alimentés artificiellement. A deux ans, la probabilité cumulée de ne pas présenter de problème de santé était de 34% chez les enfants allaités sevrés précocement et de 37% chez les non-allaités (risque relatif ajusté 1,10 ICà95% 0,87-1,38, p=0,43). La probabilité à deux ans de présenter un événement sévère était la même chez les enfants allaités sevrés précocement (15%) et non-allaités (14%) (risque relatif ajusté 1,19 ICà95% 0,75-1,91, p=0,44). Les taux de mortalité ne différaient pas entre ces deux groupes, et après ajustement sur le statut VIH de l'enfant, étaient similaires à ceux observés chez les enfants de Ditrame allaités de manière prolongée.

**Conclusions.** Nous n'avons pas trouvé de différence dans la survenue à deux ans de problèmes de santé chez les enfants allaités sevrés précocement et non allaités, et leur probabilité de survie était comparable à celle d'enfants allaités au long cours. Ces résultats soulignent la faisabilité et l’innocuité de ces interventions dans un contexte urbain africain avec accès à l’eau potable et à des conseils en matière d’alimentation infantile.
